# Supplementary material for: The interactome of intact mitochondria by cross-linking mass spectrometry provides evidence for coexisting respiratory supercomplexes
Source: Mol Cell Proteomics. 2017 Dec 8;17(2):216–32. doi: 10.1074/mcp.RA117.000470 (PMC5795388; doi:10.1074/mcp.RA117.000470)
Supplement: Supplemental Data [file supp_RA117.000470_133922_0_supp_23978_fzffwf.zip › spectra_annotation/mito_spectra_annotation/67-1-6-1-1-1.pdf]

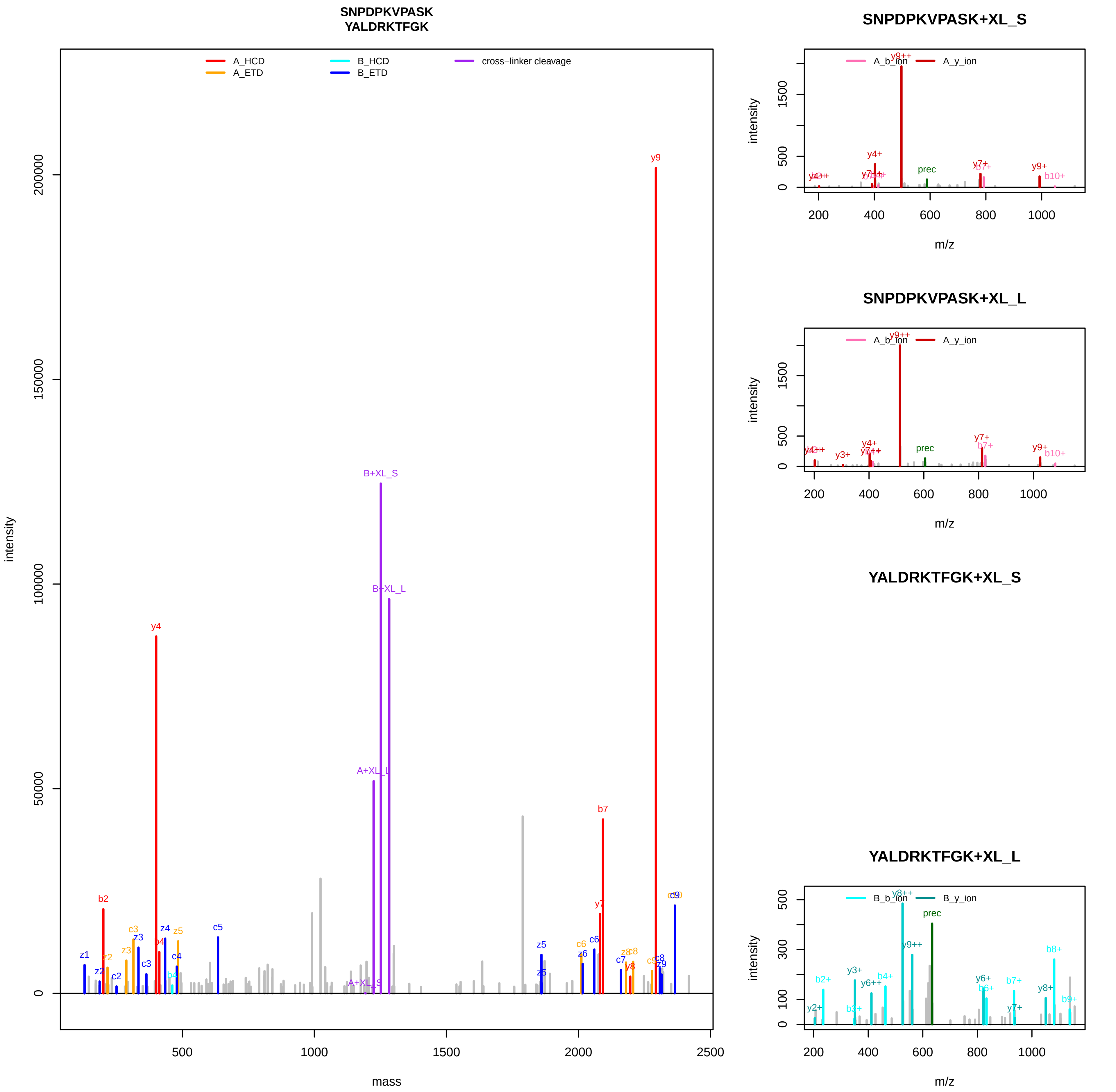
SNPDPKVPASK
YALDRKTFGK
A_HCD
A_ETD
B_HCD
B_ETD
cross−linker cleavage
intensity
mass
0
50000
100000
150000
200000
500
1000
1500
2000
2500
y9
B+XL_S
B+XL_L
A+XL_L
A+XL_S
y4
b7
b2
c3
z3
z4
z5
c5
c4
c2
z1
z2
b4
z5
z6
c6
c7
y8
c8
z8
c9
z9
SNPDPKVPASK+XL_S
SNPDPKVPASK+XL_L
YALDRKTFGK+XL_S
YALDRKTFGK+XL_L
A_b_ion
A_y_ion
B_b_ion
B_y_ion
m/z
y9++
y4+
y7+
y9+
b7+
b10+
y3+
y7++
prec
y8++
b2+
y3+
y6++
b4+
y9++
y6+
b6+
b7+
y8+
b8+
b9+
y2+
b3+
y7+
0
500
1500
300
100
200
400
600
800
1000
